# Supplementary material for: Rapid Diagnostic of Streptococcus suis in Necropsy Samples of Pigs by thrA-Based Loop-Mediated Isothermal Amplification Assay
Source: Microorganisms. 2023 Sep 29;11(10):2447. doi: 10.3390/microorganisms11102447 (PMC10608932; doi:10.3390/microorganisms11102447)
Supplement: Supplementary file 1 [file microorganisms-11-02447-s001.zip › Supplementary Data_Protocol S1-2624889_proofreaded.pdf]

Rapid Diagnostic of *Streptococcus suis* in Necropsy Samples of Pigs by *thrA*-based Loop-Mediated Isothermal Amplification Assay

Julian Hess [1], Antonia Kreitlow [2], Karl Rohn [3], Isabel Hennig-Pauka [1,*,†] and Amir Abdulmawjood [2,*,†]

[1] Field Station for Epidemiology (Bakum), University of Veterinary Medicine Hannover, Foundation 49456 Bakum, Germany; julian.hess@tiho-hannover.de

[2] Institute for Food Quality and Food Safety, University of Veterinary Medicine Hannover, Foundation 30173 Hannover, Germany; antonia.kreitlow@tiho-hannover.de

[3] Institute for Biometry, Epidemiology and Information Processing, University of Veterinary Medicine Hannover, Foundation, 30559 Hannover, Germany; karl.rohn@tiho-hannover.de

* Correspondence: isabel.hennig-pauka@tiho-hannover.de (I.H.-P.); amir.abdulmawjood@tiho-hannover.de (A.A.); Tel.: +49-511-953-7833 (I.H.-P.); +49-511-856-7440 (A.A.)

† These authors contributed equally to this work

1. Supplementary Data

1.1. Detailed Procedure for Serotyping of S. suis Isolates by In-House Multiplex PCR

Streptococcus (S.) suis isolates were serotyped using in-house PCR with modifications by Silva et al. and Kerdsin et al. [1,2]. Performing this conventional multiplex PCR, the *S. suis* species was confirmed by *gdh*-gene and differentiated by the five capsular serotypes (*cps*-types) 1, 2, 7, 9 (Silva et al., 2006) and 4 (Kerdsin et al., 2014), as well as virulence-associated factor genes *epf*, *mrp*, *sly* and *arc*A [1,2]. Each primer mix included an internal positive control. DNA was extracted from pure *S. suis* subcultures using a Tris-HCL lysis buffer (pH 8.5) and two heating steps at 60 and 95°C. Amplification is carried out in 25 µL of reaction mixture, containing 12.5 µL Qiagen Multiplex PCR Reaction Mix (Qiagen GmbH, Hilden Germany), 2.5 µL primer mix, 0.1 µL vector pGL3 (10^{-4}), 7.4 µL PCR-grade water and 2.5 µL DNA template using the Mastercycler pro S (Eppendorf AG, Hamburg, Germany). Each run included a corresponding positive and negative control. After initial denaturation (94°C, 2 min), amplification comprises 30 cycles of denaturation (94°C, 1 min), annealing (58°C, 1 min) and extension (72°C, 1:30 min) followed by a final extension at 72°C for 2 min. The amplicons were separated by gel electrophoresis in 2% agarose gel at 170V for 95 min using Thermo Scientific GeneRuler 100bp DN Ladder as DNA marker. The amplification bands were visualised and interpreted by UV detection using the AlphaImager EC with the AlphaEase Software (Biozym Scientific GmbH, Hess. Oldendorf, Germany).

1. Silva, L.M.; Baums, C.G.; Rehm, T.; Wisselink, H.J.; Goethe, R.; Valentin-Weigand, P. Virulence-associated gene profiling of Streptococcus suis isolates by PCR. *Vet Microbiol* **2006**, *115*, 117-127, doi:10.1016/j.vetmic.2005.12.013.
2. Kerdsin, A.; Akeda, Y.; Hatrongjit, R.; Detchawna, U.; Sekizaki, T.; Hamada, S.; Gottschalk, M.; Oishi, K. Streptococcus suis serotyping by a new multiplex PCR. *J Med Microbiol* **2014**, *63*, 824-830, doi:10.1099/jmm.0.069757-0.
